# Endothelial dysfunction in obstructive sleep apnea patients

**DOI:** 10.1007/s11325-021-02382-4

**Published:** 2021-05-07

**Authors:** Michał Harańczyk, Małgorzata Konieczyńska, Wojciech Płazak

**Affiliations:** 1 Department of Diagnostic Medicine, John Paul 2Nd Hospital, Prądnicka Str 80, 31-202 Kraków, Poland; 2 Department of Cardiac and Vascular Diseases, John Paul 2Nd Hospital, Jagiellonian University Medical College, Prądnicka Str 80, 31-202 Kraków, Poland

**Keywords:** CPAP, Endothelin-1, SICAM-1, Von Willebrand factor, Thrombin-antithrombin complex

## Abstract

**Purpose:**

Obstructive sleep apnea syndrome (OSAS) is an independent risk factor for cardiovascular diseases. The aim of the study was to assess the influence of OSAS on endothelial dysfunction and thrombosis biomarkers and to evaluate the effect of treatment with continuous positive airway pressure (CPAP) on biomarker levels.

**Methods:**

NT-proBNP, sICAM-1, endothelin-1, von Willebrand factor, D-dimers, and thrombin-antithrombin complex (TAT) were measured in 50 patients diagnosed with moderate-to-severe OSAS. All patients underwent transthoracic echocardiography, and 38 months after the inclusion, 16 CPAP users and 22 non-CPAP users were reassessed.

**Results:**

Sleep-related indices of apnea-hypopnea index (AHI) and mean SpO_2_ were associated with higher sICAM-1 levels (AHI < 30: 7.3 ± 4.7 vs. AHI ≥ 30: 19.5 ± 19.4 mg/ml, *p* = 0.04; SpO_2_ ≥ 90%: 11.9 ± 9.3 vs. SpO_2_ < 90%: 23.6 ± 25.8, *p* = 0.04). sICAM-1 levels were significantly higher in obese patients, particularly with BMI ≥ 40. Plasma levels of TAT were significantly correlated with the increased right ventricular size (right ventricular diameter ≤ 37 mm: 0.86 ± 0.70 vs. > 37 mm: 1.96 ± 1.20 ng/ml, *p* = 0.04). Endothelin-1 levels were higher in patients with decreased right ventricular function (right ventricle TDI-derived S′ ≥ 12 cm/s: 11.5 ± 10.9 vs. < 12 cm/s: 26.0 ± 13.2 pg/ml, *p* = 0.04). An increase in NT-proBNP was related to impaired parameters of the right ventricular contractile function. There were no correlations between long-term CPAP therapy and the levels of biomarkers.

**Conclusion:**

Severe OSAS influences endothelial damage as manifested by an increase in sICAM-1 levels. Changes in right ventricular structure and function, observed mainly in patients with higher TAT and endothelin-1 levels, are also manifested by an increase in NT-proBNP levels. Long-term CPAP treatment does not seem to influence biomarkers in patients with moderate-to-severe OSAS, which may help to explain the lack of influence of CPAP on cardiovascular risk reduction.

## Introduction

Obstructive sleep apnea syndrome (OSAS) is considered an epidemic disease in the modern world, affecting approximately 7% of men and 5% of women [1]. It is a chronic disorder characterized by recurrent episodes of upper airway collapse during sleep, resulting in hypoxia. Its effect on sleep quality and daytime sleepiness is widely acknowledged, but it has been also recognized as an independent risk factor for cardiovascular diseases such as arterial hypertension or stroke. Although the strong correlation between OSAS and cardiovascular diseases is well recognized, the detailed underlying mechanism remains unknown. Numerous possible mechanisms had been proposed to explain the association between OSAS and cardiovascular diseases, including the inappropriate supply of oxygen, abnormal sympathetic activity, increased circulating inflammatory mediators, or an imbalance of the coagulation/fibrinolysis system [2–4].

Biomarkers have been evaluated for several applications in patients with OSAS including diagnosis, prediction of disease course, and therapeutic guidance. In addition, recent studies have demonstrated a positive correlation between several biomarkers and severity of OSAS [5, 6]. Many potential OSAS biomarkers have been proposed, with pro-inflammatory and procoagulant factors being the most frequently studied. Hypoxia as a result of cyclic apnea and hypopnea episodes increases endothelin-1 levels and, as a consequence, causes vasoconstriction. Intermittent hypoxia enhances also the expression of adhesion molecules such as soluble form of intracellular adhesion molecule-1 (sICAM-1). Cell adhesion molecules enable the process of binding circulating leukocytes with the endothelial cells, which is supposed to be the primary step in the pathogenesis of atherosclerosis. As OSAS is associated with an increased cardiovascular risk and inflammation and is fundamental to the development of cardiovascular diseases, it is likely that the severity of OSAS will be related to the levels of biomarkers. Moreover, the assessment of circulating biomarkers of inflammation may become a useful tool for identifying patients with high cardiovascular risk. Consequently, it should be expected that the reduction of elevated biomarker levels will be possible through the use of effective OSAS treatment. Moreover, it has been documented that repeated episodes of nocturnal hypoxia in OSAS patients may result with a hypercoagulable state, and thus, it could be an independent risk factor for cardiovascular and cerebrovascular episodes [7]. Some studies have suggested significant correlations between OSAS and thrombin-antithrombin complex (TAT), D-dimers, and von Willebrand factor (vWF) [8, 9]. TAT is formed as a result of the inhibition of thrombin by antithrombin. D-dimers are the final products of the plasmin-mediated degradation of fibrin. D-dimers and TAT are biomarkers of coagulation activation, enabling the diagnosis of thrombotic events. vWF is synthesized in endothelial cells and megakaryocytes. It is responsible for facilitating platelet plug formation. Elevated plasma levels of vWF may indicate endothelial dysfunction and are found in several cardiovascular diseases, e.g., as pulmonary artery hypertension.

Other biomarkers linking cardiovascular diseases with OSAS are cardiac neurohormones. NT-proBNP is the first-line biomarker recommended for diagnosing heart failures, but its value in the early detection of subclinical changes in heart structures and contractility is also known [10]. NT-proBNP has been also established as a prognostic marker in heart failure, coronary artery disease, and cardiac hypertrophy [11, 12]. There are limited data about predicting OSAS-related cardiovascular events before the manifestation of clinical and echocardiographic findings. Therefore, the use of biomarkers for the detection of subclinical changes in the heart and for risk stratification has gained importance.

Similarly, mechanisms responsible for the development of structural and functional changes in the right ventricle (RV) are still controversial. The right ventricle plays a pivotal role in the morbidity and mortality of patients with cardiopulmonary disease. Several studies reported decreased RV contractility in patients with severe OSAS [13, 14]. Repeated oxygen desaturations lead to pulmonary vasoconstriction, which results in pulmonary remodeling and dysfunction in patients with OSAS. It has been recently documented that decreased RV function is independent of the presence of pulmonary hypertension and independently related to sleep apnea severity based on the apnea–hypopnea index (AHI) [15]. Therefore, early determination of subclinical RV dysfunction may be important in early diagnosis of heart failures and administration of early treatment to improve prognosis. Echocardiography is the leading method for the heart evaluation and also a good non-invasive tool to measure the increased right-heart pressures [16]. The evaluation of hemodynamics in OSAS is also clinically useful, since the negative effects of pressure changes during apnea/hypopnea episodes result in flow disturbances. As chest pressure becomes highly negative during respiration in OSAS patients, the decreased pressures according to the respiratory cycle affect pulmonary and systemic hemodynamics, mainly by increasing afterload [17].

For this reason, a group of biomarkers with a recognized role in the assessment of pulmonary circulation and inflammatory processes was selected in order to establish their levels in patients with newly diagnosed OSAS and during the treatment of patients with CPAP.

The aim of the study was (1) to determine endothelial function, thrombotic activation, and possible cardiac dysfunction in patients with newly diagnosed OSAS by means of sICAM-1, endothelin-1, D-dimers, vWF, TAT, and NT-proBNP concentrations examination; (2) to compare the levels of these biomarkers with OSAS parameters and with the structure and function of right heart assessed by echocardiography; and (3) to determine the possible changes in endothelial function and thrombotic activation in patients during CPAP treatment.

## Material and methods

**Fig. 1:**
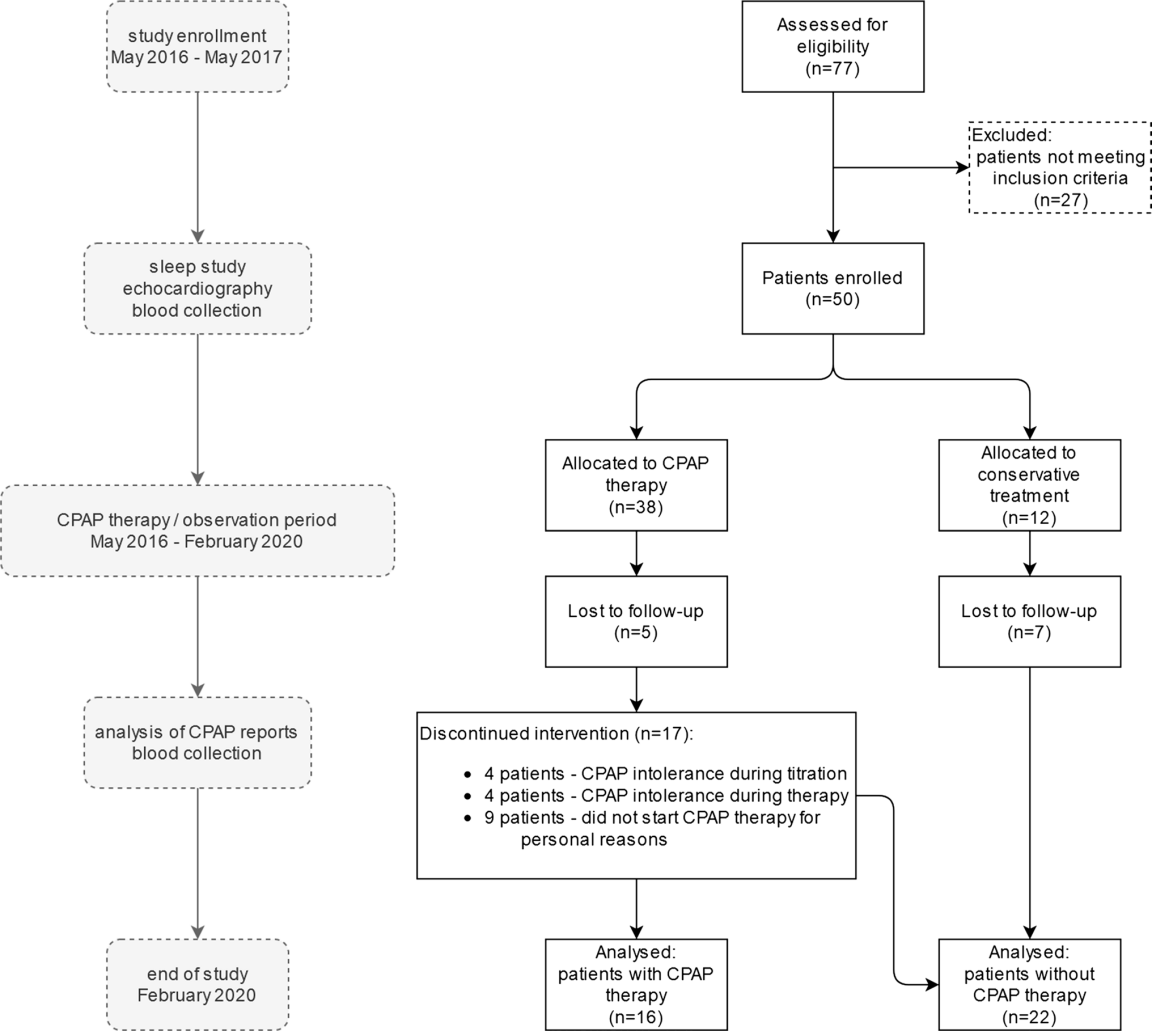
Study protocol

The study involved 77 consecutive patients admitted to hospital suffering from suspected OSAS. Men and women over the age of 18 were included. The exclusion criteria were congestive heart failure, severe valvular heart disease, severe or uncontrolled pulmonary disease (such as pulmonary hypertension, chronic obstructive pulmonary disease, or asthma), active neoplasm, or any other uncontrolled internal diseases, such as hypertension, hypothyroidism, or diabetes mellitus. The study protocol is presented in Fig. 1.

### Sleep test and CPAP adjustment

Seventy-seven patients underwent an overnight in-lab recording using a sleep-monitoring system (Embletta MPR, type III according to the American Academy of Sleep Medicine, AASM). Standard recommendations of sleep scoring criteria were applied [18]. Electrocardiography, airflow analysis, and pulse oximetry were performed. Ventilatory flows were measured with airflow cannulas fitted both over the nose and the mouth. Respiratory movements were determined using inductive plethysmography belts. Body position was monitored with the use of a built-in position sensor. Arterial oxygen saturation (SpO_2_) was measured transcutaneously with a finger pulse oximeter.

Respiratory events were scored using the 2012 AASM criteria [19]. Obstructive apnea (OA) was scored in the event of absence or reduction of the baseline airflow with continued respiratory effort to less than 10% lasting 10 s or longer. Hypopnea (H) was defined as a 30–89% reduction in the respiratory airflow amplitude lasting at least 10 s and accompanied by a decrease of at least 3% in oxygen saturation. Central apnea (CA) was scored as an absence or reduction to less than 10% of baseline airflow without continued respiratory effort, lasting 10 s or longer. When an event met apnea criteria and was associated with absent inspiratory effort in the initial part of the event, followed by resumption of inspiratory effort in the second part of the event, it was scored as a mixed apnea (MIX). The AHI was defined as the average number of episodes of apnea and hypopnea per hour. OSAS was defined as an AHI of > 5 per hour in the presence of symptoms such as daytime sleepiness. A decrease in the SpO_2_ of 3% or more from the baseline was defined as desaturation. Oxygen desaturation index (ODI) was calculated as the total number of desaturation episodes per hour.

Moderate-to-severe OSAS (AHI ≥ 15/h) was diagnosed in 50 out of 77 patients who underwent the sleep study. CPAP was recommended to all patients with AHI ≥ 30 or when the AHI was between 15 and 30 and the patient complained about severe daytime sleepiness. Therefore, CPAP therapy was not initiated in 4 patients due to the presence of moderate OSAS and the absence of significant clinical symptoms. Nine patients declined the proposed CPAP therapy or did not initiate therapy after the titration. Four patients discontinued treatment as a result of poor tolerance during CPAP titration, and 4 patients discontinued CPAP therapy after a few months due to poor treatment tolerance. The final analysis of CPAP parameters was based on the data obtained from 16 patients who had been treated with CPAP therapy during the study period. The following devices were applied: RESMED AutoSet S9 (ResMed, Bella Vista, Australia) and REMstar Auto (Philips-Respironics, Murraysville, USA) with the initial pressure set around 7–13 cmH_2_O, and initial “ramp” time, expiratory pressure relief, and a facial mask were individually adjusted. In CPAP users, all available data were downloaded from patients’ CPAP machines at the follow-up visit.

### Echocardiography

A day before the CPAP titration, the entire group of 50 patients had undergone a complete standardized two-dimensional (2-D) transthoracic echocardiography study using the Philips IE33 device (transducer X5-1; 1.3 to 4.2 MHz). Measurements of cardiac chambers and transvalvular flows were made according to the established criteria [20]. For assessment of the right atrium and ventricle, the RV-focused 4-chamber view was preferred, and mid-cavity RV (RVD) dimensions were acquired. Right ventricular systolic pressure (RVSP) was estimated using standard Doppler practices, but several views were used to determine the maximal velocity. M-mode technique was used to assess the maximum systolic excursion of the lateral tricuspid annulus (TAPSE) in an apical 4-chamber view. Tissue Doppler imaging (TDI) was performed to obtain peak systolic (S′) velocity and peak early (E′) diastolic tricuspid annular velocity.

### Laboratory methods

Endothelin-1 and sICAM-1 levels were determined using Nori® Human ICAM-1 ELISA Kit (Genorise Scientific, USA) by quantitative ELISA (enzyme-linked immunosorbent assay) in the blood serum. According to the manufacturer’s recommendations, the plasma for sICAM-1 was diluted three times. Thrombin–antithrombin complexes were assessed in citrate plasma, using AssayMax™ Human Thrombin-Antithrombin Complex ELISA Kit (Assaypro, USA). Readings were taken at a wavelength of 450 nm using an absorbance microplate reader ELx 800 (BioTek Instruments, Vinooski, VT, USA). Values were converted to concentration units of the studied parameters on the basis of the calibration curve made by KC Junior software (Bio Tek Instruments). NT-proBNP levels were determined using the Cobas e601 module (Roche Diagnostics, Risch-Rotkreuz, Swiss). Von Willebrand factor and D-dimers were determined using BCS® XP System (Siemens, Cardiff, Wales). Fasting blood samples were taken in the morning following the sleep recordings, a day before the CPAP titration started. For the second time, samples were taken during the follow-up visit.

All procedures were followed in accordance with the ethical standards of the Declaration of Helsinki, and all subjects gave their informed consent. The trial was approved by the local ethics committee (approval number 112.6120.2.2016).

### Statistical analysis

Continuous variables are presented as mean and standard deviations (SD). The Shapiro–Wilk test was used to determine if the variables were not normally distributed. Student *t*-test for continuous variables was conducted to evaluate differences between the study groups. The *p*-value of < 0.05 was considered statistically significant threshold.

## Results

The study group consisted of 50 patients with mean age 60.3 ± 10.3 years. There was no relevant difference in basic clinical parameters at baseline between the patients that during the study appeared to be CPAP users or non-CPAP users (Table 1). All patients had preserved left ventricular (LV) systolic function.

**Table 1:** Main clinical characteristics of OSAS patients

Parameter		Group 1 (CPAP users)		Group 2 (non-CPAP users)	
		Baseline (*n* = 16)	Follow-up (*n* = 16)	Baseline (*n* = 34)	Follow-up (*n* = 22)
Male sex, *n* (%)		12 (75)	12 (75)	20 (59)	12 (54)
Age (years)		57.3 ± 9.2	60.1 ± 9	61.8 ± 10.4	63.4 ± 10.8
BMI (kg/m^2^)		35 ± 4.4	35 ± 5.1	33.7 ± 6.5	32.7 ± 5.1
Sleep study results					
AHI (h^−1^)		46.3 ± 18.5	2.7 ± 2.6	33.3 ± 18.1	
OA (h^−1^)		23.6 ± 22.2	0.5 ± 0.5	14.5 ± 14	
CA (h^−1^)		1.8 ± 3.4	0.1 ± 0.2	1.2 ± 2.1	
MIX (h^−1^)		5.7 ± 6.7		2.2 ± 5.8	
H (h^−1^)		15.2 ± 9.5	1.4 ± 1.8	15.6 ± 8.4	
mean SpO_2_ (%)		90.1 ± 4		88.6 ± 13.7	
ESS		10.5 ± 6		8.9 ± 6	
Time to control (months)			38 ± 4.1		38 ± 4.3
Comorbidities					
Arterial hypertension, *n* (%)		15 (94)	15 (94)	26 (76)	16 (73)
Diabetes mellitus 2, *n* (%)		5 (31)	5 (31)	10 (29)	6 (27)
Hypercholesterolemia, *n* (%)		14 (88)	14 (88)	28 (82)	20 (91)
LVEF (%)		62.1 ± 5.4		64.4 ± 2.3	

Data expressed as mean ± SD or number (%) of patients; *SD* standard deviation, *AHI* apnea–hypopnea index, *BMI* body mass index, *CA* central apnea, *CPAP* continuous positive airway pressure, *ESS* Epworth Sleepiness Scale score, *H* hypopnea, *LVEF* left ventricular ejection fraction, *Mean SpO*_*2*_ mean blood oxygen saturation, *MIX* mixed apnea, *OA* obstructive apnea, *ODI* oxygen desaturation index

There was no significant difference in the levels of biomarkers between the patients with or without cardiovascular risk factors except for the significant influence of body weight on sICAM-1 and endothelin-1 (Table 2). D-dimers were higher in women and patients over 65 years of age. Furthermore, significant changes in RV structure and contractility were observed. The increase of RV size expressed as RVD and the decrease of RV function calculated as TDI-derived S′ were related to an increase of TAT and endothelin-1 levels, respectively (Table 3). The sleep study parameters (AHI, mean SpO_2_) were associated with sICAM-1 levels (Table 4). In patients with AHI ≥ 30, significantly higher sICAM-1 levels were found in males, females, patients aged < 60 or ≥ 60, and those with BMI < 35 or ≥ 35 (Table 5).

**Table 2:** Clinical parameters and biomarkers levels at the beginning of the study in OSAS patients (*n* = 50)

		NT-proBNP (pg/ml)	*p*	sICAM-1 (ng/ml)	*p*	Endothelin-1 (pg/ml)	*p*	vWF (%)	*p*	D-dimers (µg/L)	*p*	TAT (ng/ml)	*p*
Gender	Males	157.7 ± 569.8	ns	11.9 ± 19.8	ns	12.5 ± 16.6	ns	162.5 ± 58.1	ns	435.7 ± 497.2	0.03	1.14 ± 1.82	ns
	Females	170.3 ± 166.2		19.4 ± 44.0		18.1 ± 30.0		166.6 ± 53.7		1058.2 ± 1468.6		0.94 ± 1.31	
Age	< 65	60.5 ± 76.3	ns	13.3 ± 20.7	ns	13.7 ± 18.6	ns	152.9 ± 59.8	ns	383.4 ± 354.6	0.02	1.08 ± 1.83	ns
	≥ 65	302.7 ± 695.1		16.4 ± 40.9		15.7 ± 28.1		179.3 ± 47.6		1038.1 ± 1410.3		1.05 ± 1.39	
BMI	< 30	142.5 ± 199.6	ns	6.8 ± 3.7	0.04	3.1 ± 3.3	0.04	142.6 ± 44.6	ns	307.1 ± 145.8	ns	1.27 ± 2.20	ns
	≥ 30	168.5 ± 522.3		17.1 ± 17.4		18.2 ± 12.6		170.7 ± 58.1		748.1 ± 1096.1		1.01 ± 1.40	
BMI	< 40	181.5 ± 504.2	ns	10.5 ± 8.7	0.01	11.4 ± 7.9	0.02	160.6 ± 57.3	ns	693.1 ± 1077.1	ns	1.12 ± 1.66	ns
	≥ 40	61.2 ± 52.8		35.9 ± 32.2		30.9 ± 21.5		181.7 ± 48.4		471.8 ± 293.7		0.82 ± 1.66	
Normotensive patients		134.0 ± 229.9	ns	10.7 ± 9.4	ns	13.0 ± 21.8	ns	142.2 ± 40.4	ns	304.3 ± 138.8	ns	0.87 ± 1.05	ns
Arterial hypertension		168.4 ± 502.6		15.5 ± 33.4		14.9 ± 23.3		168.7 ± 58.3		726.6 ± 1072.5		1.11 ± 1.75	
Non-diabetic		101.7 ± 137.8	ns	17.3 ± 36.2	ns	15.3 ± 26.0	ns	163.6 ± 62.2	ns	718.4 ± 1170.7	ns	1.17 ± 1.80	ns
Diabetes mellitus		303.5 ± 822.3		8.3 ± 5.0		12.7 ± 12.0		164.8 ± 39.9		519.3 ± 369.7		0.83 ± 1.21	
Normal lipid profile		97.2 ± 119.1	ns	8.2 ± 4.1	ns	12.6 ± 17.6	ns	141.2 ± 36.3	ns	351.2 ± 208.1	ns	0.74 ± 1.43	ns
Hypercholesterolemia		174.6 ± 503.6		15.8 ± 33.2		14.9 ± 23.8		168.3 ± 58.4		717.2 ± 1074.0		1.13 ± 1.69	

*BMI* body mass index, *NT-proBNP* N-terminal part of the propeptide of BNP, *sICAM-1* soluble form of intracellular adhesion molecule-1, *TAT* thrombin-antithrombin III complex, *vWF* von Willebrand factor

**Table 3:** Functional and structural changes in the right heart and biomarkers levels at the beginning of the study in OSAS patients (*n* = 50)

		NT-proBNP (pg/ml)	*p*	sICAM-1 (ng/ml)	*p*	Endothelin-1 (pg/ml)	*p*	vWF (%)	*p*	D-dimers (µg/L)	*p*	TAT (ng/ml)	*p*
RV structural changes													
RVOT (mm)	≤ 30	99.2 ± 89.8	ns	7.5 ± 5.0	ns	10.7 ± 13.2	ns	137.4 ± 35.9	ns	797.2 ± 1693.5	ns	0.53 ± 0.93	ns
	> 30	182.5 ± 529.8		17.1 ± 34.7		15.9 ± 25.1		170.3 ± 59.0		600.8 ± 639.5		1.14 ± 1.79	
RVD (mm)	≤ 37	220.7 ± 592.4	ns	15.5 ± 34.4	ns	15.2 ± 26.4	ns	164.6 ± 51.4	ns	836.7 ± 1244.1	ns	0.86 ± 0.70	0.04
	> 37	65.5 ± 74.6		16.2 ± 24.4		13.4 ± 15.1		168.5 ± 63.8		460.5 ± 340.1		1.96 ± 1.20	
RAA (cm^2^)	≤ 20	110.2 ± 114.6	ns	16.7 ± 36.8	ns	14.6 ± 27.3	ns	158.3 ± 46.9	ns	795.6 ± 1279.4	ns	0.90 ± 1.66	ns
	> 20	246.5 ± 655.0		13.2 ± 22.3		14.6 ± 16.6		173.4 ± 65.0		499.2 ± 574.8		1.25 ± 1.64	
RV functional changes													
TAPSE (mm)	≥ 24	69.5 ± 52.2	ns	12.4 ± 22.8	ns	11.3 ± 16.3	ns	164.5 ± 45.6	ns	498.4 ± 578.9	ns	0.86 ± 1.41	ns
	< 24	241.2 ± 623.6		16.5 ± 36.1		17.3 ± 27.2		163.6 ± 64.6		789.8 ± 1235.0		1.25 ± 1.82	
RV S’ (cm/s)	≥ 12	68.9 ± 61.2	0.01	14.1 ± 29.2	ns	11.5 ± 10.9	0.04	159.3 ± 51.8	ns	676.3 ± 1023.4	ns	1.00 ± 1.63	ns
	< 12	466.8 ± 559.1		18.6 ± 37.6		26.0 ± 13.2		184.4 ± 69.7		656.8 ± 869.1		1.47 ± 1.69	
TRPG (mmHg)	≤ 25	87.4 ± 94.5	ns	21.0 ± 43.2	ns	18.4 ± 30.8	ns	153.8 ± 55.6	ns	745.3 ± 1307.2	ns	1.38 ± 2.01	ns
	> 25	305.9 ± 634.8		9.0 ± 5.8		10.8 ± 9.7		190.4 ± 56.0		727.4 ± 585.7		0.84 ± 1.18	

*NT-proBNP* N-terminal part of the propeptide of BNP, *RAA* right atrial area, *RVD* mid-cavity right ventricular diamete, *RVOT* right ventricular outflow tract, *sICAM-1* soluble form of intracellular adhesion molecule-1, *S′* peak early systolic tricuspid annular velocity, *TAPSE* tricuspid annular plane systolic excursion, *TAT* thrombin-antithrombin III complex, *TRPG* tricuspid regurgitant peak gradient, *vWF* von Willebrand factor

**Table 4:** Sleep study parameters and biomarkers levels at the beginning of the study in OSAS patients (*n* = 50)

			NT-proBNP (pg/ml)	*p*	sICAM-1 (ng/ml)	*p*	Endothelin-1 (pg/ml)	*p*	vWF (%)	*p*	D-dimers (µg/L)	*p*	TAT (ng/ml)	*p*
AHI	(h^−1^)	< 30	106.8 ± 84.8	ns	7.3 ± 4.7	0.04	8.9 ± 11.7	ns	159.4 ± 62.2	ns	722.9 ± 1309.4	ns	1.13 ± 1.22	ns
		≥ 30	199.2 ± 595.9		19.5 ± 19.4		18.3 ± 27.5		167.1 ± 52.2		605.5 ± 705.8		1.13 ± 1.89	
mean SpO_2_	(%)	≥ 90	196.2 ± 536.0	ns	11.9 ± 9.3	0.04	14.5 ± 18.8	ns	162.9 ± 61.5	ns	653.3 ± 1084.4	ns	1.27 ± 1.79	ns
		< 90	65.2 ± 51.7		23.6 ± 25.8		14.9 ± 32.5		168.9 ± 38.4		657.9 ± 718.7		0.52 ± 0.98	

*AHI* apnea–hypopnea index, *mean SpO*_*2*_ mean blood oxygen saturation, *NT-proBNP* N-terminal part of the propeptide of BNP, *sICAM-1* soluble form of intracellular adhesion molecule-1, *TAT* thrombin-antithrombin III complex, *vWF* von Willebrand factor

**Table 5:** sICAM-1 levels in subgroups of patients differentiated according to gender, age or BMI

		Males	Females	< 60 years	≥ 60 years	BMI < 35	BMI ≥ 35	
AHI (h^−1^)	< 30	6.9 ± 4.5	7.8 ± 5.3	6.2 ± 2.0	7.9 ± 5.7	7.0 ± 4.9	8.5 ± 4.5	
	≥ 30	14.6 ± 11.9	30.8 ± 30.5	19.8 ± 14.4	19.2 ± 23.3	14.9 ± 13.5	24.6 ± 24.5	
	*p*	0.04	0.03	0.03	0.04	0.04	0.03	

*AHI* apnea–hypopnea index, *BMI* body mass index, *sICAM-1* soluble form of intracellular adhesion molecule-1

The mean follow-up period was 38 ± 4.2 months. All patients using CPAP achieved a significant reduction in AHI as shown in Table 1. In our study, the mean duration of CPAP use was 3 ± 2.3 h/night (all-day analysis), and CPAP usage mean time during days with device usage was 4.7 ± 2.1 h/night, according to CPAP machines’ readings. The analysis of studied biomarkers in CPAP users and non-CPAP users showed no significant change during the observation (Table 6).

**Table 6:** The levels of biomarkers in CPAP users and non-users at baseline and at follow-up

			CPAP users			non-CPAP users	
		Baseline (*n* = 16)	Follow-up (*n* = 16)	*p*	Baseline (*n* = 34)	Follow-up (*n* = 22)	*p*
NT-proBNP	pg/ml	257.5 ± 806.9	122.0 ± 93.5	ns	103.2 ± 82.9	115.8 ± 170.7	ns
sICAM-1	ng/ml	8.31 ± 5.6	8.1 ± 5.2	ns	14.3 ± 23.6	13.0 ± 16.3	ns
Endothelin-1	pg/ml	11.3 ± 12.7	6.8 ± 6.8	ns	16.9 ± 21.7	18.0 ± 30.0	ns
D-dimers	µg/L	659.1 ± 870.9	635.8 ± 940.5	ns	730.3 ± 1319.7	480.6 ± 296.8	ns
vWF	%	172.6 ± 72.8	191.5 ± 117.5	ns	149.4 ± 43.1	156.8 ± 43.9	ns
TAT	ng/ml	0.87 ± 2.05	0.53 ± 1.40	ns	1.45 ± 1.55	0.99 ± 0.99	ns

*CPAP* continuous positive airway pressure, *NT-proBNP* N-terminal part of the propeptide of BN, *sICAM-1* soluble form of intracellular adhesion molecule-1, *TAT* thrombin-antithrombin complex, *vWF* von Willebrand factor

## Discussion

The main finding of the study was that a high apnea–hypopnea index correlates significantly with higher sICAM-1 levels. Correspondingly, it was shown that higher sICAM-1 levels are present in patients with mean saturation < 90%. Similarly, an increase in NT-proBNP as an indicator of impaired RV contractile function measured by TDI-derived S′ was demonstrated. The other interesting finding is a decreased level of endothelin-1 in a subgroup of CPAP users with a baseline BMI ≤ 35. However, the study did not reveal any significant changes in the levels of observed biomarkers as a result of CPAP treatment.

## Biomarkers in OSAS

### NT-proBNP

NT-proBNP analysis in our study provided the assumed conclusion that its values tend to be higher with age. The study also demonstrated an increase in NT-proBNP in patients with impaired parameters of RV contractile function (statistically significant for RV S′ and the trend for TAPSE) and structural function (the trend for right atrial area, RAA). Moreover, we found that NT-proBNP tends to be higher in patients with high AHI. This corresponds well with other studies, which found no statistical difference in baseline values of NT-proBNP [21].

A further analysis of sleep study parameters in our patients revealed a tendency to higher NT-proBNP values in patients presenting more episodes of apnea, which was confirmed also by Kohno et al. [22]. This finding extends the recent data obtained by Kulkas et al. [23] who claimed that since apneic episodes arise as a consequence of the complete upper-airway collapse, they result in a more pronounced drop in blood saturation level. Consequently, they may impose a more serious pathophysiologic impact than hypopneas which results from the incomplete collapse of the upper airway. Indeed, apneas may be regarded as more significant than hypopneas when assessing the related impact on the long-term cardiac risk.

### Endothelial dysfunction markers

In our study, sICAM-1 levels were significantly higher in patients with BMI ≥ 40. Moreover, endothelin-1 levels were higher in patients with decreased right ventricular function. Overall, our data support the concept that the severity of OSAS might be related to endothelial dysfunction. Cyclic changes in the breathing pattern causing episodes of apnea and hypopnea result in hypoxemia and hypercapnia. Hypoxia increases endothelin-1, a potent vasoconstrictor with proinflammatory properties. Hypoxia also enhances the expression of adhesion and is involved in the induction of endothelial and myocyte apoptosis. Since endothelial dysfunction has been demonstrated in several cardiovascular disorders, potential common pathophysiological pathways appear to influence the morbidity of OSAS patients [24].

As presented in this study, a high level of AHI, corresponding to the effect of sleep apnea on the body, correlates significantly with the levels of sICAM-1. Similarly, a higher sICAM-1 levels were confirmed in our patients for mean saturation < 90%. This finding supports the thesis that endothelial damage is manifested by an increase in the levels of pro-inflammatory molecules as a result of repeated desaturation during sleep [25].

Our data confirm the results of the previously published study by Carratu et al. [26] who claimed that endothelin-1 levels were significantly higher in obese patients. Similarly, it was determined that the difference was more strongly associated with weight than the severity of sleep apnea.

Consistent with such a conclusion is the significant relationship between the degree of obesity and the levels of sICAM-1 found in our and other studies [27]. Several studies demonstrated that plasma levels of adhesion molecules are increased among obese individuals, whereas weight reduction was associated with a decrease in ICAM levels [28]. ICAM levels are elevated in obese individuals, even in potentially healthy individuals, but this also applies to patients diagnosed with OSAS [29]. In our study, there is a clear tendency towards an increase of sICAM-1 with increasing body mass index (BMI) in patients with BMI ≥ 40.

As presented in our study, high BMI is a strong factor increasing the levels of endothelin-1. Therefore in obese patients, the levels of endothelin-1 did not decrease, because BMI did not change during the observation period in our study. However, in a subgroup of CPAP users with a baseline BMI ≤ 35, endothelin-1 decreased significantly.

The results of our study indicate that patients with AHI > 30 (OSAS grade 3) tend to have higher endothelin-1 levels. This value does not reach statistical significance, which is most likely due to the size of the study group. By contrast, Ursavaş et al. [30] found that OSAS can increase the circulating levels of adhesion molecules independently of BMI, smoking status, or cardiovascular disease, although the analysis also included patients with mild OSAS.

Our findings suggest that RV systolic dysfunction is associated with higher levels of endothelin-1. Our study shows significantly lower RV TDI-derived S′ values, but a trend was also observed for TAPSE values. The increase in endothelin-1 levels may be the result of persistent nocturnal hypoxia, thereby leading to an increase in the vascular resistance in pulmonary vessels. This may be followed by pulmonary arteriolar vasoconstriction, which could adversely affect RV function. It is to note that RV function is substantially influenced by RV afterload, which is mainly determined by pulmonary vascular resistance and slightly influenced by preload. As a result, we observed RV damage expressed as a decrease in RV contractility parameters. Consistently one could expect an increase of RVSP; however, it is not confirmed by this study. The explanation might be the fact that the sensitivity of RVSP estimation by echocardiography is suboptimal. Consequently, imprecise results might follow, particularly in situations when significant tricuspid jet cannot be visualized or there is a suboptimal Doppler Wave angle, or poor acoustic window, which is frequent in OSAS patients [31].

### Coagulation biomarkers

Our study shows that plasma levels of TAT were significantly correlated with the increased right ventricular size. Furthermore, significantly higher levels of D-dimer in women and patients ≥ 65 years were observed. Nonetheless, there was no significant difference in coagulation biomarkers between OSAS and patients with AHI 15–30 or ≥ 30, and those levels remained unchanged after CPAP treatment. This conclusion is in line with the available data from Zamaron et al. [32] and von Känel et al. [33]. It should be emphasized that single studies have shown that an increased level of pro-thrombotic factors in OSAS patients is related to other comorbidities (e.g., hypertension) [34].

### Biomarkers in CPAP

Our study had a long follow-up period, and there was a significant reduction in AHI in all CPAP patients which applied to each category of sleep events. Nevertheless, no significant changes in the levels of observed biomarkers (NT-proBNP, sICAM-1, endothelin-1, vWF, D-dimers, TAT) were revealed as a result of CPAP treatment.

CPAP therapy remains the gold standard of OSAS treatment, although the effects of long-term treatment on clinical parameters are not fully convincing [35]. The available data confirm the effect of CPAP on the reduction of daytime sleepiness, decreased number of road accidents, and the improvement of blood pressure control. On the other hand, the impact of CPAP on cardiovascular events such as heart attacks or heart failures remains controversial [36]. Similarly, the recent analysis of randomized clinical trials shows no difference in all-cause mortality, major adverse cardiovascular events, or cardiovascular death [37]. Some argue that poor protection against cardiovascular outcomes is a result of the low level of use, which may not be sufficient to achieve the therapeutic effect of CPAP. However, the sensitivity analyses and the subgroup analyses in this meta-analysis did not reveal consistent results concerning the impact of longer CPAP use (≥ 4 h per night) on cardiovascular outcomes in OSAS patients [37].

In our observation, the level of CPAP use met the criteria of the recommended minimum of 4 h per night, but the total number of days when the device was applied was not satisfactory. Therefore, low adherence of patients as a probable cause of the lack of significant differences in biomarker levels is considered. The short time of follow-up duration, proposed as another factor affecting the vascular outcome, seems not to be the case in our study as the average time from the inclusion to follow-up visit was 1138.8 ± 125 days (38 ± 4.2 months).

In accordance with the results, Svatikova et al. [21] and Çifçi et al. [38] demonstrated that CPAP therapy does not affect NT-proBNP. On the contrary, Tasci et al. [39] showed a reduction in NT-proBNP after CPAP treatment, but this study differed in the time at which NT-proBNP was measured (first day after CPAP treatment initiation). The presented data do not conclude on the role of CPAP and its impact on the potential reduction of NT-proBNP, which in a situation of preexisting comorbidities such as heart failures, is well established [40].

The expected reduction of endothelial dysfunction markers was not observed in our study; however, according to some researchers, numerous pathophysiological pathways can be reversed using effective CPAP therapy, and many studies report a beneficial effect of CPAP on endothelial dysfunction markers. The Ohga study [41] showed a reduction in sICAM-1 levels in patients using nasal continuous positive airway pressure (nCPAP) after 8–18 months of therapy. Another study showed sICAM-1 reduction, which was most pronounced in patients with severe obesity [29].

We have shown that among non-CPAP users, endothelin-1 levels do not change significantly. However, patients treated with CPAP tended to have lower endothelin-1 levels than non-CPAP users. We speculate that the differences may become more apparent with a higher number of studied patients. These results are in line with Zamarrón et al. study [32], in which after 1 year of CPAP treatment, a significant decrease in circulating levels of sICAM-1 was found, but none in vWF or endothelin-1 levels. Moreover, our data are consistent with those reported by Grimpen et al. [42], who found significant changes of endothelin-1 neither during sleep nor in the first night on CPAP therapy, nor under 14 months CPAP treatment, and with Diefenbach et al. [43] who stated that endothelin-1 levels of untreated OSAS patients and patients under effective long-term (> 6 months) treatment with nCPAP were within the normal range and were not elevated when compared with healthy subjects. Furthermore, Turnbull et al. [44] found that the levels of endothelin-1 also have not changed in patients treated with CPAP for a year after temporary discontinuation of treatment. These results contradict the conclusions of recent meta-analysis [45], although the authors admit that the studies included in the analysis were highly heterogeneous.

Among procoagulant molecules vWF, D-dimer, and TAT were selected, due to their role in the coagulation system, as well as the contradictory findings from previous studies assessing their impact on OSAS. In previous studies, the short-term evaluation did not reveal any significant impact of CPAP on vWF and D-dimer levels [33], similar conclusions can be drawn from the randomized study with sham-CPAP [8]. Another short observation revealed changes in nocturnal and early morning levels of vWF, whereas D-dimer levels remained unchanged [46]. Some researchers have reported hypercoagulability in patients with OSAS as manifested by elevated TAT levels, but similarly, no change in this parameter was observed after 1 month of CPAP therapy [34].

### Limitations

Non-CPAP users were a mixed group of patients, 5 of whom were referred at the outset for conservative treatment and 17 who did not start or discontinued CPAP for different reasons, even though they met the treatment criteria. Therefore, comparisons with the non-CPAP user group are limited by their heterogeneity. The determination of the structure and function of the right ventricle by echocardiography might be somehow limited. Moreover, no other measurement of right heart structures was attempted. Cardiac magnetic resonance (CMR) remains the gold standard of RV assessment, although the advantages of echocardiography, such as low costs of examination, possibility to perform the examination in patients regardless of implanted pacemakers, or claustrophobia, play a significant role in everyday clinical practice. As portable devices remain useful in the diagnosis of OSAS in patients with a high pretest likelihood of having moderate-to-severe OSAS, full polysomnography was not performed [47]. Finally, our study population was relatively small but proportional to comparable studies performed in OSAS patients.

## Conclusions

Severe OSAS influences endothelial damage as manifested by an increase in sICAM-1 levels. Changes in the right ventricular structure and function, observed mainly in patients with higher TAT and endothelin-1 levels, are also manifested by an increase in NT-proBNP levels. Long-term CPAP treatment does not seem to influence biomarker levels in patients with moderate-to-severe OSAS, which may help to explain the lack of influence of CPAP on cardiovascular risk reduction.
